# Burnout in United States Healthcare Professionals: A Narrative Review

**DOI:** 10.7759/cureus.3681

**Published:** 2018-12-04

**Authors:** Thomas P Reith

**Affiliations:** 1 Internal Medicine, Medical College of Wisconsin, Milwaukee, USA

**Keywords:** burnout, wellness, well-being, stress, fatigue

## Abstract

Burnout has reached rampant levels among United States (US) healthcare professionals, with over one-half of physicians and one-third of nurses experiencing symptoms. The burnout epidemic is detrimental to patient care and may exacerbate the impending physician shortage. This review gives a brief history of burnout and summarizes its main causes, effects, and prevalence among US healthcare workers. It also lists some strategies that physicians, organizations, and medical schools can employ to counter the epidemic.

## Introduction and background

What is burnout?

Broadly speaking, burnout is a combination of exhaustion, cynicism, and perceived inefficacy resulting from long-term job stress. It was first described in 1974 by the clinical psychologist Herbert Freudenberger, who often volunteered at a free clinic in the then drug-ridden East Village of New York City. Over time, Freudenberger observed emotional depletion and accompanying psychosomatic symptoms among the clinic’s volunteer staff. He called the phenomenon “burnout,” borrowing the term from drug-addict slang. Freudenberger defined burnout as exhaustion resulting from “excessive demands on energy, strength, or resources” in the workplace, characterizing it by a set of symptoms including malaise, fatigue, frustration, cynicism, and inefficacy:

There is a feeling of exhaustion, being unable to shake a lingering cold, suffering from frequent headaches and gastrointestinal disturbances, sleeplessness and shortness of breath. … The burn-out candidate finds it just too difficult to hold in feelings. He cries too easily, the slightest pressure makes him feel overburdened and he yells and screams. With the ease of anger may come a suspicious attitude, a kind of suspicion and paranoia. The victim begins to feel that just about everyone is out to screw him. … He becomes the ‘house cynic.’ Anything that is suggested is bad rapped or bad mouthed. … A sign that is difficult to spot until a closer look is taken is the amount of time a person is now spending in the free clinic. A greater and greater number of physical hours are spent there, but less and less is being accomplished. He just seems to hang around and act as if he has nowhere else to go. Often, sadly, he really does not have anywhere else to go, because in his heavy involvement in the clinic, he has just about lost most of his friends [1].

In addition, Freudenberger noted that burnout often occurred in contexts requiring large amounts of personal involvement and empathy, primarily among “the dedicated and the committed.”

Over the next decade, the social psychologist Christina Maslach built upon Freudenberger’s work. At the University of California, Berkeley, Maslach developed a model of burnout consisting of three dimensions: emotional exhaustion, depersonalization, and a diminished sense of personal accomplishment [2]. In 1981, she proposed the Maslach Burnout Inventory (MBI), which consists of three subscales to measure the extent of an individual’s symptoms along each dimension [2]. The MBI remains the most commonly used instrument to assess burnout to this day [3].

Why should we care about burnout?

The consequences of burnout are not limited to the personal well-being of healthcare workers; many studies have demonstrated that provider burnout is detrimental to patient care. For example, the number of major medical errors committed by a surgeon is correlated with the surgeon's degree of burnout [4] and likelihood of being involved in a malpractice suit [5]. Among nurses, higher levels of burnout are associated with higher rates of both patient mortality [6] and dissemination of hospital-transmitted infections [7]. In medical students, burnout has been linked to dishonest clinical behaviors, a decreased sense of altruism [8], and alcohol abuse [9]. High rates of physician burnout also correlate with lower patient satisfaction ratings [10].

At an institutional level, burnout results in greater job turnover and increased thoughts of quitting among physicians [11] and nurses [12]. It also results in decreased workforce efficiency: a recent Mayo Clinic study estimated the loss of productivity due to physician burnout as the equivalent of eliminating seven entire medical school graduating classes [13]. Consequently, burnout may contribute to an already impending physician and nursing shortage.

## Review

How prevalent is burnout?

Attending Physicians

Over half of physicians in the United States (US) experience symptoms of burnout, a rate nearly double that of workers in other professions after controlling for hours worked, age, sex, and other factors [14]. Furthermore, burnout among physicians has shown signs of increasing. The 2013 Medscape Lifestyle Report – based on the surveyed responses of over 20,000 physicians – reported a nationwide burnout rate of 40% [15], yet the 2017 Report found a rate of 51% [16], representing a 25% increase in four years. Another recent study supports the Medscape findings, reporting a 9% increase in burnout between 2011 and 2014 [17]. Physicians working the front lines of care (emergency medicine, family medicine, internal medicine and obstetrics/gynecology (OB/GYN)) are at especially high risk for burnout, and female physicians are more likely to experience burnout than their male colleagues [16].

Nurses & Physician Assistants

Burnout is not limited to physicians. A 2001 study found that 43% of nurses working at US hospitals experience symptoms of emotional exhaustion [18], and a 2011 study reported burnout prevalences of 37% among nurses providing direct patient care in nursing homes, and 33% among hospital nurses [19]. While burnout in physician assistants is less studied, initial reports suggest it may be similarly high [20].

Residents & Medical Students

Burnout is especially prevalent among physicians in training. A 2016 study of residents of all specialties at a tertiary academic center reported an overall burnout rate of 69%, with a 78% rate among surgical residents and a 66% rate among non-surgical residents [21]. A 2009 review supports these findings, reporting overall rates of resident burnout up to 75% [22]. In medical students, burnout levels are not much better. A 2013 review estimated that at least half of students at US medical schools experience symptoms [23], and a 2018 meta-analysis of over 16,000 students worldwide found that 44% suffered from burnout [24].

What causes burnout?

Although burnout is caused by a myriad of factors, surveys of physicians have helped to identify common themes. As part of its annual Physician Lifestyle Report, Medscape gives physicians a list of possible burnout causes and asks them to rank their significance. Over the last five years, “too many bureaucratic tasks (e.g., charting, paperwork),” “spending too many hours at work,” and “increasing computerization of practice (electronic health records (EHRs)),” have consistently been ranked as three of the top four factors [15, 16].

Too Many Bureaucratic Tasks

Today’s physicians spend a large amount of time on documentation required for a growing number of quality programs initiated by Medicare, Medicaid, and private insurance companies. Such programs cause burnout by impeding physicians from spending time with their patients [17]. On average, US physicians spend 2.6 hours per week complying with external quality measures; in an outpatient setting, this is enough time to see approximately nine additional patients [25]. Moreover, for each hour of clinical face time that physicians spend with patients, an additional two are consumed by administrative and clerical work [26]. The former president of the American Medical Association (AMA), Robert M. Wah, attempted to summarize the collective feelings of US physicians in the following statement:

Physicians want to provide our patients with the best care possible, but today there are confusing, misaligned and burdensome regulatory programs that take away critical time physicians could be spending to provide high-quality care for their patients [27].

Too Much Time at Work

The average US physician works 51 hours per week, with one quarter of US physicians working more than 60 hours per week [28]. When surveyed by the AMA, one half of physicians responded that they would prefer to work fewer hours [29]. Inverse correlations have been found between hours worked and job satisfaction. Physicians working in specialties requiring more hours report lower job satisfaction, and physicians working in specialties requiring fewer hours report higher job satisfaction [30].

Increasing Computerization of Practice

When EHRs were first introduced, they were touted as a way to streamline workflows and reduce the clerical burden on physicians. In this respect, however, EHRs have had the opposite effect of creating more work. In one recent study, primary care physicians spent nearly six hours out of an 11.4-hour workday on EHR tasks, including around 1.5 hours at night after the clinic was closed [31]. Such tasks included documentation, order entry, billing and coding, and inbox management. Put another way, physicians spent more time in the EHR than they did treating patients. In a recent interview, Steven Strongwater, CEO of Massachusetts-based Atrius Health, summarized the impact of EHRs on Atrius’s physicians as follows:

The electronic medical record has clearly added work to a physician’s day, and people who are so dedicated and committed are working late into the evenings in what we would call ‘pajama time.’ In general, what seems to happen is that our docs will work during the day — they’ll work a full day, sometimes 8 or 10 hours or longer — they’ll go home for a brief period of time, and then they’ll get back on their record in order to finish the work of the day that evening [32].

Is burnout a distinct disorder?

The validity of burnout as an independent diagnosis remains controversial. While the majority of studies use the MBI for measurement, the scales and cutoff values employed are often arbitrary. Indeed, one recent review concludes that the measurement of burnout in the literature is so heterogenous that it is impossible to conclude anything about its prevalence [3]. Another criticizes the MBI as being “neither grounded in firm clinical observation nor based on sound theorizing” [33]. A third calls it “unrealistic”:

The three-dimensional structure of the burnout syndrome is unrealistic [and] the mere fact of defining burnout as job-related is not nosologically discriminant. … The arbitrariness surrounding the elaboration of the MBI constitutes a fundamental problem, especially given the central role of the instrument in the definition of the burnout phenomenon [34].

Furthermore, the symptoms of burnout seem to overlap with those of depressive disorders. In one study, over 90% of participants assessed as “burned out” by the MBI also met diagnostic criteria for depression and scored 15 or greater on the Patient Health Questionnaire-9 (PHQ-9) [35]. In another study, depressed and “burned out” participants displayed similar attentional and behavioral alterations [36].

Burnout is also not recognized in the 5th edition of the *Diagnostic and Statistical Manual of Mental Disorders (DSM-5)*, the official classification of psychiatric disorders in the United States [37].

How can we combat burnout?

Regardless of burnout’s nosological classification, an epidemic of unhappy and demoralized physicians seems worthy of acknowledgement. Unfortunately, there remains a relative paucity of evidence on how to address the problem. Still, recent research indicates that efforts at both the individual and organizational levels can prove effective; indeed, the best way forward likely involves a combination of the two [38]. To that end, major health organizations have begun developing guidelines aimed at decreasing burnout and increasing well-being. Last year, the Mayo Clinic described nine strategies that, when implemented, resulted in a 7% decrease in burnout over a two-year period [39]. In April 2018, a number of physician educators and wellness experts published a Charter on Physician Well-being [40], which presents guiding principles that individuals and groups should use when addressing burnout. Some suggestions for addressing burnout are listed below.

Involve Leadership

There is an ancient saying that the fish begins to stink at the head. In other words, problems within any organization often stem from its executive leadership. Evidence suggests that greater leadership qualities in physician supervisors decreases burnout and increases job satisfaction among the physicians they oversee [41]. Consequently, healthcare administrators must acknowledge burnout as a systemic problem and promote a culture of self-care among their employees, starting from the top down. To help accomplish this, some hospitals, such as Stanford and Mount Sinai, have created the administrative position of chief wellness officer [42]. If leadership is inadequate, however, organizations must be willing to make changes. In most companies, the board of directors has no problem ousting a CEO who is not delivering profits. Similarly, a healthcare executive overseeing a majority of unhappy physicians may need to be replaced.

Choose Incentives Wisely

Many healthcare systems motivate physicians with financial rewards, either adjusting physicians’ salaries based on productivity (i.e., revenue generation) or handing out performance-based bonuses [43]. Yet productivity-based compensation often leads to overwork and/or shortening the time spent per patient, which in turn leads to increased burnout. Such consequences are by no means a modern phenomenon. In *The Wealth of Nations*, the 18^th^ century economist Adam Smith offered the following warning:

Workmen, … when they are liberally paid by the piece, are very apt to overwork themselves, and to ruin their health and constitution in a few years [44].

To avoid these problems, organizations may want to consider performance-independent salary models or offer alternative rewards such as greater schedule flexibility or time off [39]. They may also want to incorporate measures of well-being into performance assessments [40].

Encourage a Work-Life Balance

Physicians often find it difficult to balance long hours at work with their personal lives. Organizations can help mitigate this problem by allowing physicians to work fewer hours in exchange for reduced compensation, or by granting them greater flexibility. For example, physicians could choose to start the work day earlier or later, or work longer hours on certain days and shorter hours on others. Organizations can also let physicians devote more time to their favorite aspect of work (e.g., patient care, education, administration, or research). Physicians who spend at least 20% of their time on the part of work they find most fulfilling significantly lower their chances of burning out [45]. On an individual level, physicians can work to improve their time management skills. Eliminating time used inefficiently at work allows more time to be spent at home.

Encourage Peer Support

Recent years have seen diminished personal interaction between physicians. Increased documentation requirements and the rise of EHRs have caused physicians to spend increasing amounts of time on computer systems. Moreover, doctors’ lounges – where physicians historically relaxed and discussed cases – have disappeared from many hospitals, resulting in a loss of camaraderie and an increased sense of isolation [42]. Yet evidence suggests that encouraging physician solidarity reduces burnout: when Mayo physicians engaged in one hour of small group discussions every other week, they experienced significant reductions in depersonalization and emotional exhaustion [46]. One small way for hospitals to promote physician interaction might be providing coffee and snacks at gathering spots analogous to the office “water cooler.” Recently, Stanford has taken a more creative approach, paying for small groups of doctors to dine together at local restaurants [42].

Furnish Resources for Self-care and Mental Health

Mental health remains a taboo subject among physicians, and many are reluctant to pursue treatment due to potential shame, income loss, or licensure actions [47]. Organizations can counter this stigma by helping physicians seek treatment in ways that minimize repercussions. Examples include extending the hours of confidential mental health services to include times that physicians are not at work, and/or providing coverage to allow physicians to attend appointments [40]. Organizations can also furnish resources encouraging individual physicians to practice self-care; examples include offering healthy food in cafeterias, providing mindfulness or exercise programs at the hospital or clinic, and facilitating memberships to local gyms. Furthermore, physicians can be equipped with protected time to devote to these practices.

Target Burnout from Day One of Medical School

Finally, burnout must be addressed from the onset of medical training. This problem cannot be addressed at the resident and attending levels if students are already burned out by the time they get there. Recent efforts addressing burnout at select schools have been met with success. Vanderbilt University's School of Medicine has implemented a wellness program where students promote healthy habits by holding each other accountable [48]. At Northwestern University's Feinberg School of Medicine, second-year medical students are tasked with improving their self-care by choosing a personal health behavior to change and tracking their progress towards it [49]. Perhaps the most drastic changes, however, have been made at the Saint Louis University (SLU) School of Medicine, where the curriculum has been redesigned over the past decade to reduce student stressors and “produce a less toxic educational environment.” Changes included the implementation of a pass/fail grading system, reducing unnecessary detail in coursework, and introducing electives throughout the preclinical years. As a result, SLU students experienced reductions in depression, stress, and anxiety while maintaining similar levels of academic performance [50].

## Conclusions

Burnout has emerged as a major problem plaguing 21^st^ century American medicine. If not addressed, the burnout epidemic may continue to worsen, to the detriment of patients and physicians alike. Experts have identified good starting points to confront this problem, and it is time for healthcare institutions nationwide to put their suggestions into practice.
